# Selective progesterone receptor modulators (SPRMs): progesterone receptor action, mode of action on the endometrium and treatment options in gynecological therapies

**DOI:** 10.1080/14728222.2016.1180368

**Published:** 2016-05-14

**Authors:** Andrea Wagenfeld, Philippa T.K. Saunders, Lucy Whitaker, Hilary O.D. Critchley

**Affiliations:** ^a^ Bayer HealthCare, Drug Discovery, TRG Gynecological Therapies, Berlin, Germany; ^b^ MRC Centre for Inflammation Research, The University of Edinburgh, Edinburgh, UK; ^c^ MRC Centre for Reproductive Health, The University of Edinburgh, Edinburgh, UK

**Keywords:** Cofactors, endometrium, HMB, PAEC, selective progesterone modulator

## Abstract

**Introduction**: The progesterone receptor plays an essential role in uterine physiology and reproduction. Selective progesterone receptor modulators (SPRMs) have emerged as a valuable treatment option for hormone dependent conditions like uterine fibroids, which have a major impact on women’s quality of life. SPRMs offer potential for longer term medical treatment and thereby patients may avoid surgical intervention.

**Areas covered**: The authors have reviewed the functional role of the progesterone receptor and its isoforms and their molecular mechanisms of action via genomic and non-genomic pathways. The current knowledge of the interaction of the PR and different SPRMs tested in clinical trials has been reviewed. The authors focused on pharmacological effects of selected SPRMs on the endometrium, their anti-proliferative action, and their suppression of bleeding. Potential underlying molecular mechanisms and the specific histological changes in the endometrium induced by SPRMs (PAEC; **P**rogesterone receptor modulator **A**ssociated **E**ndometrial **C**hanges) have been discussed. The clinical potential of this compound class including its impact on quality of life has been covered.

**Expert Opinion**: Clinical studies indicate SPRMs hold promise for treatment of benign gynecological complaints (fibroids, heavy menstrual bleeding; HMB). There however remains a knowledge gap concerning mechanism of action.

## 1. Introduction: the impact of progesterone and uterine function

Progesterone is a steroid hormone that plays a key role in development, differentiation, and normal functioning of female reproduction-related target tissues including the uterus (endometrium and myometrium), the ovary, and the mammary gland as well as in the regulation of the hypothalamic–pituitary–gonadal axis. Abnormal progesterone responses are implicated in a wide spectrum of benign human reproductive disorders, including fibroids, endometriosis and adenomyosis, abnormal uterine bleeding (AUB; including heavy menstrual bleeding [HMB]), and miscarriage.[1–4] From the onset of puberty to menopause, progesterone is mainly produced by the corpus luteum in the ovary with smaller amounts secreted by the adrenal glands.

Actions of progesterone on the female reproductive system are primarily mediated by progesterone receptors (PRs) synthesized from a single gene (*PR)* and expressed as two main protein isoforms (PR-A, PR-B).[5,6] Beyond its prominent function in reproductive tract tissues, progesterone is also involved in regulation of cellular functions in the central nervous system [7] influencing reproductive behaviors. Progesterone also plays an important role during pregnancy and has striking impacts on the function of the breast.[8]

The uterine endometrium comprises of epithelial cells (lining the luminal surface and glands), stromal cells, immune cells, and blood vessels and it is arranged in two morphologically and functionally distinct zones, the inner basal zone and the outer functional zone with the latter being shed at menstruation.[6,9] The human myometrium, localized between endometrium and perimetrium, is a heterogeneous tissue and can also be subdivided in two zones, namely the outer myometrium and a functionally distinct inner myometrial layer called the uterine junctional zone.[10,11] This ‘junctional’ zone can be visualized with magnetic resonance imaging (MRI) but is not histologically distinct. The myometrium is largely made up of smooth muscle cells but also contains connective tissue, blood vessels, and immune cells. The principal uterine cellular targets for progesterone, expressing PR-A and PR-B, are the epithelial and stromal/decidual cells in the endometrium [12,13] and smooth muscle cells in the myometrium.[14]

During the menstrual cycle, the human endometrium undergoes dynamic changes including proliferation, differentiation, tissue breakdown, and shedding (menstruation) in response to fluctuating peripheral concentrations of ovarian-derived estrogen and progesterone. Estrogens, acting via their cognate receptors, play a key role in modulating tissue function in the follicular (proliferative) phase by inducing epithelial and stromal cell proliferation leading to a thickened functional zone. Estrogens also stimulate expression of *PR,* thus ensuring progesterone responsiveness in the post-ovulatory luteal (secretory) phase.[6] Estrogen levels decline after ovulation [15,16] and rising concentrations of progesterone secreted by the corpus luteum initiate a differentiation program characterized by growth and coiling of the spiral arteries, secretory transformation of the glands, an influx of distinct immune cells, especially specialized uterine natural killer cells, and transformation of the stromal fibroblasts (decidualization) in preparation for blastocyst implantation.[17,18] Progesterone induces genes that allow the endometrium to permit embryo attachment and directly controls vascular permeability.[6,19]

## 2. PRs

### 2.1. Intracellular and membrane PR isoforms

The PR as a member of the nuclear hormone receptor superfamily is a ligand-dependent transcription factor [20,21] characterized by structural motifs like the N-terminal A/B region, a highly conserved DNA-binding domain (DBD), a hinge region, and a C-terminal ligand-binding domain (LBD). The DBD is composed of two conserved zinc fingers that distinguish nuclear receptors from other DNA-binding proteins.

The PR-A and PR-B mRNA isoforms are both transcribed from the same *PR* gene and the proteins they encode are identical in their DNA-binding and ligand-binding properties; it is likely that PR A/B homodimers and heterodimers exist.[22] PR-B (116 kDa) differs from PR-A (94 kDa) only by an additional stretch of 165 AA at the N-terminus of the protein. A marked physiological difference is the action of PR-A as a trans-dominant inhibitor of PR-B [23] and it even exerts this inhibitory action onto other members of the NR superfamily including ER, androgen receptor (AR), MRI, and GR.[24]

Differential recruitment of PR [25,26] and associated transcriptional co-regulators to gene promoters are critical to tissue selective impacts of progesterone (details see below) for example, whereas in the uterus progesterone stimulates growth of leiomyomas, it inhibits growth of the endometrium.[6]

**Figure 1.**
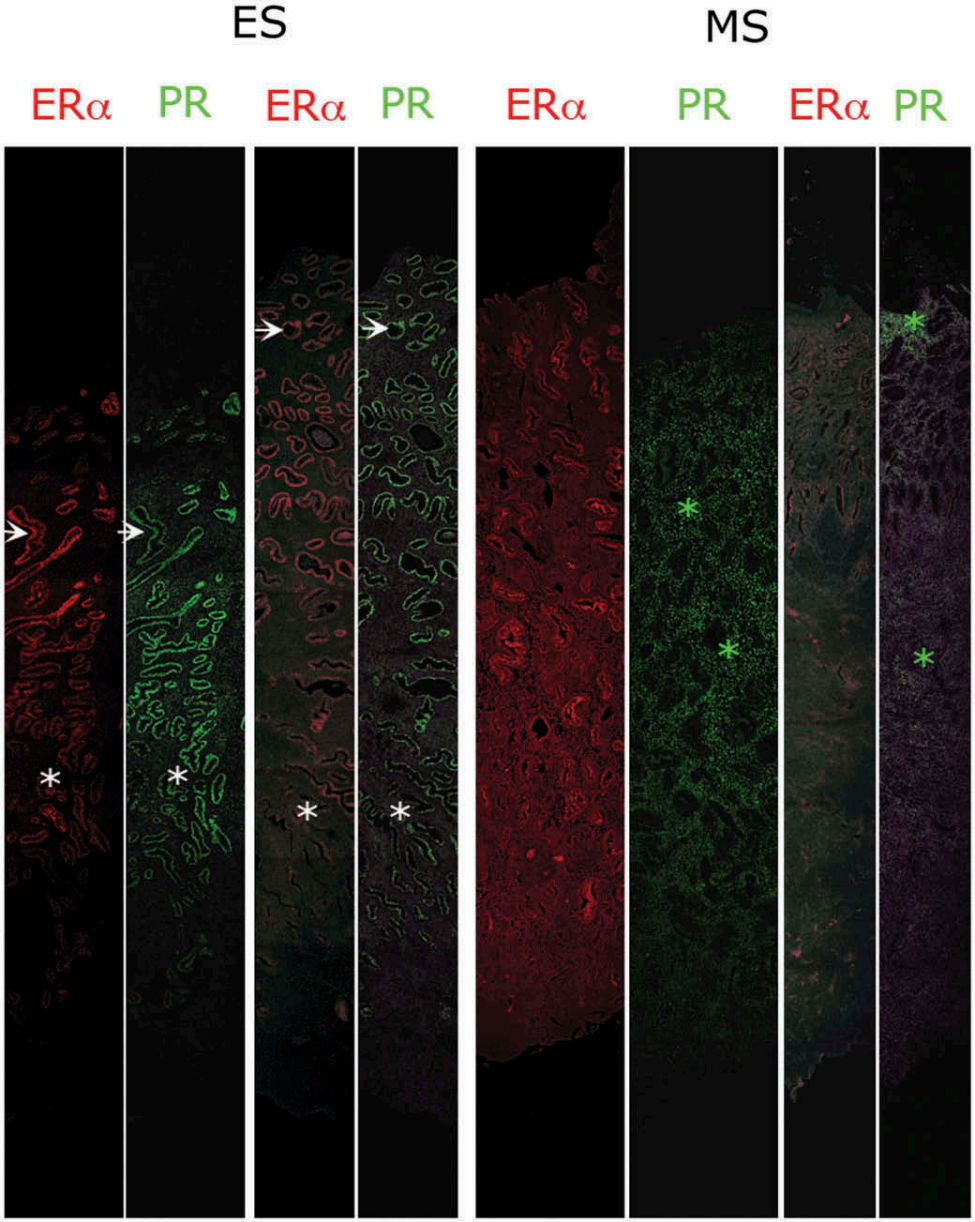
Immunolocalisation of ERα and PR in full thickness sections of human endometrium. Images shown from four samples of uterine tissue recovered during the early (ES) or mid (MS) secretory phases of the cycle: each section represents the full thickness of the uterine wall with the lumen at the top and myometrium at the bottom. Sections were co-stained for ERα (red) and PR (green) using standard protocols [32] for clarity the images recorded in the different channels (red, green) are shown side-by-side rather than overlaid. Note that during the ES there is intense immunopositive staining for both ERα and PR-A in the glandular epithelium in both the functional (arrows) and basal (white asterisks) layers. During the MS immunoexpression in the epithelium is down-regulated but expression of PR in stromal fibroblasts is maintained (green asterisks). Full color available online.

The ratio of PR-A and -B expression varies from tissue to tissue and is dependent on the hormonal status of the cell.[27] In full thickness sections of the human uterus, parallel expression of ERα and PR can be detected using immunofluorescence (Figure 1; antibody for PR, recognizing both A and B isoforms), demonstrating intense immunoexpression in glandular epithelium at the start of the secretory phase following induction during the follicular phase with subsequent downregulation in the mid-secretory phase. Whereas PR-A levels in epithelial cells decline in late secretory phase, PR-B levels remain constant, suggesting that this subtype may be involved in the control of glandular secretion.[6] Studies to assess the cellular localization of PR-A and PR-B have to be interpreted with caution as they are technically limited due to the common sequence of PR-A and PR-B and the abundance of PR-A and its relative level to PR-B may only be determined in a semiquantitative manner at best. PR-A appears to be predominant subtype in the stromal cells, with a less obvious decline in expression during the luteal phase than in the epithelium, which might reflect the need for prolonged progesterone PR-A signaling in this compartment to support the establishment of pregnancy.[12,28] Both receptors have been detected in leiomyoma (fibroid) tissue [29] and seem to be increased in leiomyoma compared with normal myometrium from the same patients.[30,31]

To delineate the individual roles of the receptor subtypes *in vivo,* PR isoform-specific knockout (KO) mice have been generated.[33,34] In these studies, female mice with a global ablation of both receptor subtypes failed to reproduce due to defects in both ovulation and implantation.[33,34] Specific ablation of PR-A alone also resulted in severe abnormalities in ovarian and uterine function leading to female infertility.[35] In contrast, PR-B-specific KO mice had normal ovarian and uterine responses to progesterone but exhibited reduced mammary ductal morphogenesis and alveologenesis during pregnancy. Both PR subtypes of receptor appear to mediate anti-inflammatory actions of progesterone on the endometrium.[36] Notably, both subtypes mediate progesterone-dependent responses through activation of different subsets of genes with those regulated by PR-A being both necessary and sufficient for reproductive fertility functions, while PR-B-dependent gene activation, at least in mice, plays a key role in mammary development.[24,36] Although some additional variant PR mRNAs (PR-C, -M, -S, and -T) have been described, a recent comprehensive protein analysis did not support their translation *in vivo*.[37]

In addition to the PR receptors that are members of the superfamily of transcription factors, a membrane-bound PR (mPR) first described in fish has been implicated in rapid non-genomic actions (see below) of progesterone.[38,39] Upon progesterone binding, mPRs may influence the activity of several signaling pathways, including mobilization of intracellular Ca^2+^, activation of mitogen-activated protein kinase (MAPK) cascades, and inhibition of cAMP production.[40] The physiologic relevance of the membrane PR is still unclear since their capacity to bind progesterone is relatively low compared to nuclear PRs and some studies could not even detect evidence of activation by progesterone.[41]

### 2.2. Genomic and non-genomic activation of PR

In the reproductive tract, it is likely that progesterone exerts its effects via both genomic *and* non-genomic actions mediated via PR-A or PR-B that subsequently converge to produce tissue- and cell-specific responses.[42] In the ‘classical’ genomic mode of action binding of a ligand within the LBD, conformational changes are initiated, chaperone proteins dissociate and the PR translocates to the nucleus. Within the nucleus, ligand-bound PRs interact with the transcriptional machinery and bind as homo- or heterodimers to specific *cis*-acting PR response elements (PRE), typically located in the promoter regions of target genes. Robust, specific modulation of gene transcription, however, requires recruitment of additional co-regulatory proteins to a transcription complex that includes the DNA-bound receptor. The different co-regulatory factors are generally considered to act to enhance transcription (coactivators) or to decrease the level of transcriptional activation (corepressors).[26] Over 300 co-regulators are reported to interact with PR, and it is the tissue-specific expression of the factors that orchestrates the impact of progesterone on expression of different sets of genes within target tissues.[43]

It is now well established that binding of agonists or antagonists to distinct amino acid residues within the LBD of PR alters the conformation of the receptor protein resulting in recruitment of differing type(s) of co-regulatory proteins into the transcription complex. For example, following binding of agonists, coactivators capable of modifying core histone protein side chains via acetylation or methylation are recruited and the resultant change in histone proteins facilitates access of the transcription machinery. PR coactivators include members of the steroid receptor coactivator (SRC) family (SRC-1–3) and receptor-interacting protein 140.[44] The importance of the SRC PR interactions has been elucidated by studies in KO mice demonstrating that steroid receptor coactivator-1 (SRC-1) is the primary coactivator of PR in the uterus but SRC-3 is important in the mammary gland.[45] Lately, a new class of PR modulators the Kruppel-like factors (KLFs) [46] have been described and the absence of some KLFs in distinct pathologies (e.g. KLF9 and KLF11 in endometriosis and leiomyoma) suggests roles for multiple KLFs in maintaining homeostasis in female reproductive tissues.

On the other hand, PR can also interact with corepressors and this generally occurs in the presence of ligands like mifepristone that act as antagonists. Crystal structures have shown binding of the nuclear receptor corepressor (NCoR), and the silencing mediator of retinoic acid and thyroid hormone receptor (SMRT) to both PR-A and PR-B in the presence of asoprisnil, a synthetic PR receptor modulator that is a mixed agonist/antagonist and downregulates expression of PR in endometrium.[47,48] In the human endometrium, the expression of both PR isoforms and their corepressors (NCoR and SMRT) has been observed [28,49] and is modulated over the course of the menstrual cycle in a compartment-specific manner [49] demonstrating the potential for stage-dependent gene repression.

Finally, additional cell-specific impacts of ligand-activated PR may be determined by the co-recruitment of coactivators and a number of additional transcription factors also expressed in endometrium some of which appear to play a key role in decidualization of stromal cells in preparation for establishment of pregnancy. Examples include members of the forkhead-box O (FOXO) protein family (FOXO1, FOXO3a), signal transducer, and activator of transcription (STAT5) and CCAAT enhancer-binding protein (C/EBPβ).[50]

Ligand-bound nuclear PR receptors can also be transcriptionally active at endogenous promoters lacking a canonical PRE. Transcription of these genes seems to be facilitated through nuclear protein–protein interactions with other DNA-binding transcription factors such as NFκB,[51] SP1, and AP-1.[52] Levels of gene transcription can also be modulated by post-translational modification of PRs primarily through N-terminal phosphorylation, acetylation, SUMOylation, and ubiquitination.[53,54] These modifications alter PRs trafficking, transcriptional activity, and target-gene selectivity.[55] Studies in cancer cells have shown that modifications involve phosphorylation through mitogenic protein kinases, CDK2, CK2, or MAPK of the receptor.[56] PR has been shown to trigger Src-dependent phosphorylation signaling cascades like proliferative Ras/Raf/MEKK/MAK kinase pathway.[7] The relevance for uterine tissue dysfunction remains poorly understood.

In addition to its direct effect on transcription, progesterone has been identified to influence the activity of many other signaling pathways by non-genomic (extranuclear) mechanisms in the cytoplasm.[57] These rapid non-genomic effects triggered by progesterone binding to membrane-bound receptors exhibit an onset within seconds to minutes (see Table 1) and are insensitive to transcription or translation inhibitors. A detailed description of non-genomic progesterone effects in different target tissues and potential membrane receptors involved has been reviewed by Gellersen et al.[42]

**Table 1.** Non-genomic signaling pathways reported to be triggered by progesterone.

Involved signaling pathways	
**SRC/ERK/MAPK pathway** Delayed P-dependent neuroprotection mediated by Src-ERK signaling Cyclin D1 gene induction by PR activation of the Src/MAPK pathway Rapid activation of Src/Erk1/2 and PI3K/Akt pathways in breast cancer and endometrial stromal cells via crosstalk between PR and ERα/β	Boonyaratanakornkit et al.,[58] Cai et al.,[59] Ballare et al.,[60] Mani et al. [7]
**PI3K/Akt/NFκB pathway** Stimulation mPR by P activates the PI3K/Akt/NFκB pathway resulting in (1) inactivation of FOXO transcriptional activity and (2) downregulation of miR-29c which triggers KLF4	Vares et al. [61]
**MEK1/2 and PKA** Activation of macrophages by P via mPR causes pro-inflammatory shift in mRNA expression profile and significant upregulation of cyclooxygenase 2, Il1B, and TNF and downregulation of mPRα MEK1/2 and PKA are involved in mPR signaling	Lu et al.,[62] Mani et al. [7]
**PKC** Rapid increase in basal PKC activity in VMN by P	Balasubramanian et al. [63]
**Calcium and calmodulin kinase II** P-activation of CaMKII basal activity in VMN	Balasubramanian et al. [64]
**PKG** PgRMC1 shown to mediate rapid progestin actions in various tissues (including brain) by potential activation of PKG	Bashour et al. [65]

PKA: Protein kinase A; PKC: protein kinase C; VMN: ventromedial nucleus; PKG: protein kinase G; PgRMC1: progesterone receptor membrane component 1.

Finally, the distinction between the rapid non-genomic kinase activation and genomic actions has become less certain. For example, results using breast cancer cells have demonstrated activated kinases can be recruited together with the phosphorylated nuclear PR into an integrated PRE-containing promotor.[66] According to this model, rapid signaling may be a concurrent pathway integrated into the activation of the transcriptional machinery by nuclear PR,[67] but further studies are required to validate this in nonmalignant cells.

## 3. Selective progesterone receptor modulators

### 3.1. Modulation of PR activity by selective progesterone receptor modulators

**Figure 2.**
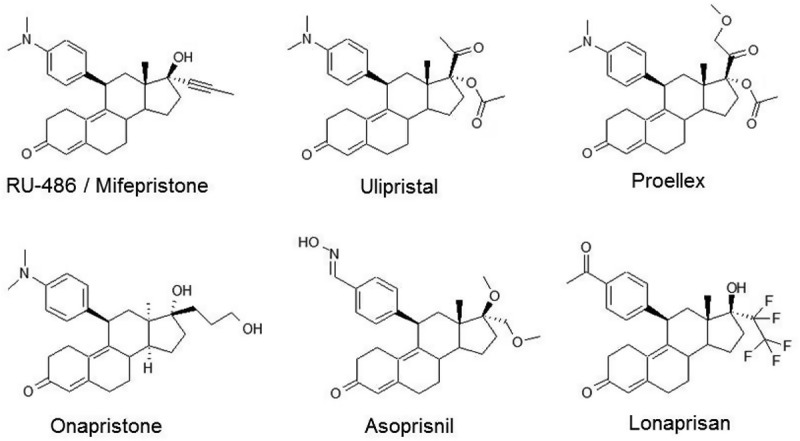
Structure of common SPRMs. Chemical structures of selective progesterone receptor modulators (SPRMs) in current clinical use or which have been in clinical development.

Selective progesterone receptor modulators (SPRMs) represent a new class of synthetic steroids, which can exert agonist, antagonist, or mixed effects on various progesterone target tissues *in vivo* upon PR binding [68] (see Figure 2). They have many potential clinical applications in female reproduction and gynecological therapies like uterine fibroids but also in the treatment of some tumors.[57]

X-ray crystal structures of the PR bound to SPRM ligands revealed that the mode of binding differs between different molecules and depends on the agonistic or antagonistic nature of the interaction.[69] The resulting effect on target genes appears to depend both on the cell type and availability of different co-regulators [70] also confirmed by evaluation of protein–protein interactions with PR and SPRMs.[71]

The first and one of the most widely used SPRMs with mixed agonistic/antagonistic properties and tissue-specific effects is mifepristone (RU486). Wardell et al. described the involvement of the amino-terminal domain of the PR and the phosphorylation of a serine for the partial agonist features of mifepristone.[72]

With an *in vitro* chromatin transcription system that recapitulates PR-mediated transcription *in vivo*, Liu et al. [44] have determined the molecular basis by which mifepristone regulates transcription in a cell-type-specific manner. Specifically, agonist-bound PR interacts only with coactivators such as SRC-1, whereas mifepristone-bound PR binds to both the coactivator SRC-1 and the corepressor silencing mediator for retinoid and thyroid hormone receptor (SMRT) so that the precise impact in different cell types may be influenced by the relative availability/abundance of these factors.

Afhuppe and colleagues [73] compared different SPRMs and their ability of interaction with PR and co-regulators. Mifepristone, onapristone, and lonaprisan (ZK230211) differ in their induced interactions of PR with the NCoR. PR modulators with marked PR agonistic activity demonstrate induced interactions with the LX-H10 peptide (contains the LxxLL motif of coactivators) similarly to the ones observed with R5020 (promegestrone), whereas SPRMs with antagonistic behavior like lonaprisan do not show any recruitment of the LX-H10 peptide.[74] In contrast to mifepristone, asoprisnil mediates the recruitment of coactivators to the PR *in vitro*. However, none of these compounds has a progesterone-like ability to oppose estrogen in the rat endometrium,[47] again demonstrating the high degree of complexity of the system as a whole.[70] There have been several recent reviews that provide informative summaries of the effects of SPRM administration.[75–77]

### 3.2. Impact of SPRMs upon leiomyoma growth and endometrial morphology

The mechanisms of fibroid growth reduction have been addressed in several *in vitro* studies but a clearer picture may be expected in the near future when fibroid biopsies from the different clinical studies using SPRMs are fully analyzed. So far, there is strong evidence that SPRMs induce apoptosis through activation of the tumor necrosis-related apoptosis-inducing ligand (TRAIL) pathways.[78] Fibroids treated with ulipristal acetate (UPA) revealed upregulation of caspase 3 and downregulation of BCL2.[79] Furthermore, ample evidence exists that strong expression of extracellular matrix in fibroids is reduced by SPRMs due to suppression of collagen synthesis (type I and III) and modulation of extracellular matrix enzymes like MMPs and TIMPs.[80]

An interesting aspect in the pathomechanism of fibroid growth has recently been addressed by Bulun et al..[81] They describe a paracrine pathway that may mediate progesterone-derived growth of leiomyoma tissue. Treatment of mature myometrial cells with estrogen and progesterone resulted in secretion of wingless type (WNT) ligands, translocation of β-catenin in neighboring leiomyoma stem-progenitor cells, and activation of gene expression critical for fibroid growth and proliferation. The importance of WNT/β-catenin signaling in formation of leiomyoma-like tumors and fibrogenesis has been shown in mice that express a constitutively active form of β-catenin in mesenchymal cells of the uterus.[82]

In this context, it is noteworthy to mention that the mediator complex subunit 12 (MED12) gene, which has been previously demonstrated to regulate β-catenin/WNT signaling, has mutations in exon 2 in nearly 70% of uterine leiomyomas.[83] The role(s) for such signaling pathways in the endometrium of women with fibroids has to date not been determined.

The administration of all family members of the SPRM class of compound has been found to date to be accompanied with morphological changes within the endometrium described as PR modulator-associated endometrial changes (PAEC).[84] These histological changes are recognized as a distinct histological entity and should not be confused with endometrial hyperplasia. SPRMs have been shown to induce a specific endometrial antiproliferative effect and the endometrial glandular epithelium shows reduced mitotic activity compared to the proliferative phase. Furthermore, evidence is accumulating that PAEC rapidly regress on cessation of treatment, although the rate of regression can be variable.[85] Whilst PAEC are now well described and appear reversible, the mechanisms by which these develop are poorly understood.

UPA is a SPRM licensed in Europe for preoperative treatment of moderate-to-severe symptoms of uterine fibroids in adult women of reproductive age and also for intermittent treatment of moderate-to-severe symptoms of uterine fibroids in adult women of reproductive age.[86] In common with other SPRMs, UPA significantly reduces menstrual bleeding and fibroid volume.[85,87]

The mechanisms responsible for these effects remain poorly understood. In keeping with other SPRMs, despite progesterone antagonism and maintenance of circulating estradiol levels, hyperplasia does not occur with any increased frequency although extensive cystic glandular dilatation is seen.[88,89] Although *in vitro* work describes antiproliferative and proapoptotic effects on leiomyoma cells,[90] there are only limited data on the effects of SPRMs upon human endometrium.

Studies in nonhuman primates (macaques) have shown a suppressive effect specifically upon the endometrium with a reduction in proliferation markers and upregulation of the AR consistent with the ‘class effect’ of other SPRMs.[91] In a long-term oral toxicity study with UPA,[92] findings in the endometrium were similar to SPRM-associated endometrial changes described in SPRM-treated women. No adverse effects were observed that would raise concerns about potential premalignancy. Detailed human *in vivo* data still remain limited to small studies. Apoptosis indices are increased in the endometrium, but knowledge of impact upon proliferation and sex steroid receptor expression to date are again limited.

Thus far, there have been no reports of cytological atypia accompanying SPRM administration in the presence of a normal endometrial biopsy prior to drug therapy with SPRMs. Administration of low-dose mifepristone (RU486; 2–5 mg) for 120 days was observed to reduce endometrial proliferation markers.[93] Administration of another SPRM with mixed agonist–antagonist activity, asoprisnil, had no reports of endometrial hyperplasia with administration limited to daily administration for 12 weeks. Oral daily doses of both 10 or 25-mg asoprisnil had no impact on markers of endometrial proliferation.

The discovery of the ‘endometrial antiproliferative effect’ of SPRMs was an important milestone in their development. This effect was initially observed in rabbit and primate endometrium. The finding of endometrial atrophy induced by the SPRM class of compound was not anticipated. The compounds were reported not to bind the estrogen receptor due to PR antagonist activity and thus the endometrium would have been expected to exhibit unopposed estrogenic effects and yet functional antiestrogenic effects were reported. The believed unique endometrial effects of SPRMs are specific to menstruating primates such as Old World monkeys and humans. Cynomolgus and rhesus macaques are good models, as their endometrium is similar to the human with respect to hormonal regulation and morphological changes during the menstrual cycle. Studies in nonhuman primates have been reported to show that SPRM administration in both spayed and intact macaques induced endometrial atrophy with stromal compaction and inhibited mitotic activity. These effects were observed following administration of mifepristone (RU486), ZK 230 211, and ZK 137 316.[94] Studies with ZK 137 316 in the rhesus monkey also showed a dose-dependent degradation of the spiral arteries in the basal layer of the endometrium. It is notable that these profound morphological changes were observed in the presence of follicular phase estrogen levels. This functional antiestrogenic effect appears to be limited to the endometrium. The oviduct and vagina are reported to be unaffected, thereby providing evidence for an ‘endometrial antiproliferative effect’ and provides support that SPRMs may target the endometrium directly and this effect may possibly be via the endometrial vasculature. Dosage of SPRM administration may be important as some effects may be dose-dependent.[94,95]

**Figure 3.**
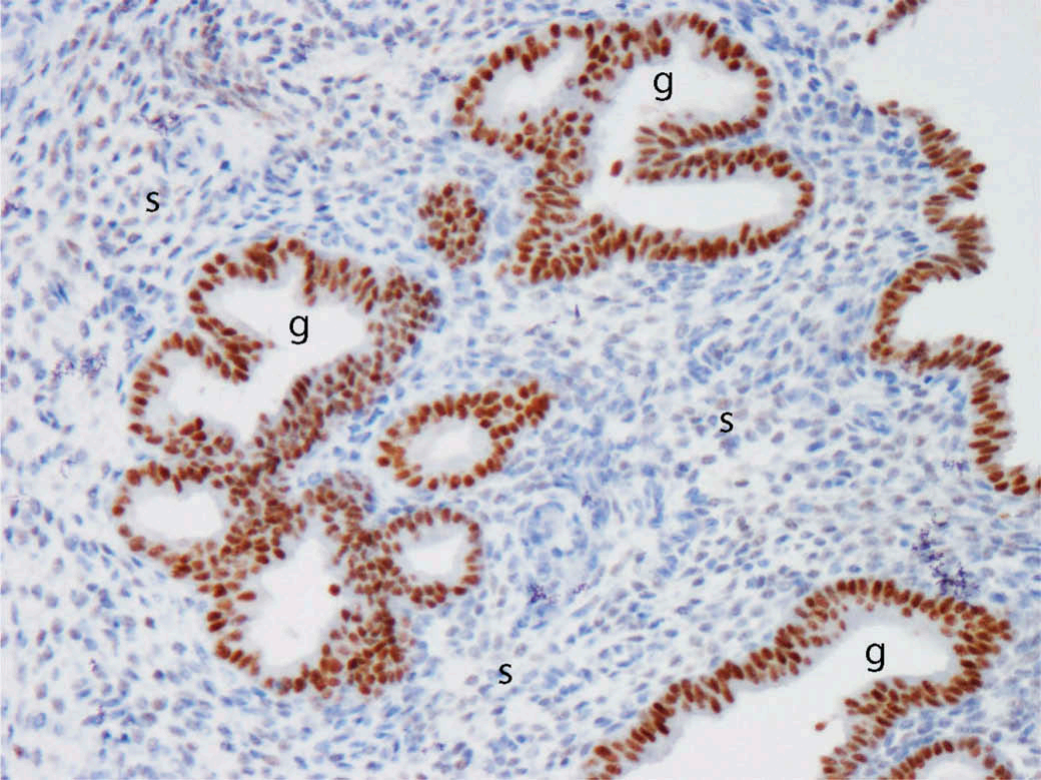
Image of progesterone receptor (PR) immuno-reactivity in human endometrium after administration of a selective PR modulator (SPRM). Note intense positive (brown) immunostaining in the glandular epithelium (g) and virtual absence of immuno-reactivity in the stroma (s). Image kindly provided by Professor Alistair Williams, University of Edinburgh. Full color available online.

The SPRM, asoprisnil, suppresses endometrial bleeding and administration has a striking histological effect on the endometrial spiral arteries which develop an unusual appearance as prominent aggregations due to abnormally thick muscular walls.[96] When global endometrial gene expression in asoprisnil-treated versus control women was performed, a most interesting and statistically significant reduction of inflammatory genes has been reported.[48] The IL-15 pathway, known to play a key role in uterine NK cell development and function, was identified at the center of a pathway analysis and suppression of IL-15 by asoprisnil was also observed on mRNA level. Furthermore, immunostaining for the uterine NK cell marker CD56 revealed an impressive reduction in the asoprisnil-treated endometrium.[48] In the normal cycling endometrium, IL-15 levels are progesterone-responsive. In the study of asoprisnil-treated endometrium, there is a downregulation of stromal PR expression, upregulation of glandular PR expression (see Figure 3), and a marked reduction in number of uterine NK cells. These observations with administration of a SPRM have provided support for a role for the IL-15 pathway in the complex interplay between endometrial stromal cells, uterine NK cells, and spiral arteries and an effect on both physiological and HMB.[48,97,98]

## 4. Clinical potential for SPRMs in management of benign gynecological disorders

Benign gynecological complaints such as HMB, fibroids, pelvic pain, and endometriosis have a very significant impact on quality of life and represent a large health-care burden. Each year in the United Kingdom, one million women seek help for HMB [99] and endometriosis has a prevalence of 2–10% of women of reproductive age.[100] There are a multitude of etiologies that can cause HMB.[101] Many cases have uterine fibroids that disrupt everyday life and fibroids remain the leading indication for hysterectomy.[102,103] While there may be relief from HMB during pregnancy and lactation, and an end to the problem at menopause, women affected will tend to suffer the adverse impacts of HMB over what should be the prime years of their lives. This can accumulate to a lifetime loss of 5–7 years of ‘healthy’ life.

Along with the direct impact on the woman and her family, there are significant costs both to the economy and the health service. In the USA, lost work-hour costs are estimated between $1.55 and 17.2 billion annually and direct costs of $4.1–9.4 billion.[104]

Current medical therapies often either fail to fully resolve symptoms or are associated with unacceptable side effects. As a result, many women opt for a definitive solution consequently hysterectomy remains the solution with the highest long-term patient satisfaction and cost-effectiveness.[99] Many women however wish to avoid surgery and to retain fertility. This is pertinent given that nearly half of all UK-born babies are to women aged 30 or over.[105] Furthermore, those from low-income backgrounds are less likely to receive a surgical treatment [106] and as such may be further penalized by ineffective medical treatment. Finally, the recent RCOG HMB audit reported that at 1-year post-referral only 35% of all women (including those given surgery) were ‘satisfied’ (or better) at the prospect of current menstrual symptoms continuing, as currently experienced, for the next 5 years.[106] There is thus a very substantial unmet need for long-term medical therapies that are effective, affordable, and without unwanted side effects.

The evidence of the impact of SPRMs on endometrial cell proliferation (reviewed above) and data from nonhuman primate studies have identified these compounds as offering significant potential for longer term medical therapy of HMB. Specifically, two members of this class, mifepristone and asoprisnil, have been shown to significantly reduce menstrual blood loss in association with fibroids but do not increase proliferation.[107,108] The impact of SPRM administration on menstrual bleeding in women without fibroids is not known.

The medical management of endometriosis is currently largely dependent upon administration of progestogens and estrogen deficiency has to date restricted long-term use of GnRH analogs. With each of these commonly used therapies, management is frequently limited by accompanying side effects and symptom control may be suboptimal. Studies with SPRM administration undertaken in women with endometriosis indicate potential clinical utility for symptom relief.[109,110] For example, mifepristone administration (50 mg for 6 months) in patients with endometriosis has been reported to have a significant effect on symptoms and extent of disease.[110] In a randomized, placebo-controlled study, asoprisnil (5, 10, or 25 mg) was administered for 12 weeks to women with a laparoscopic diagnosis of endometriosis who complained of moderate or severe pain. A significant reduction in non-menstrual pelvic pain and dysmenorrhea compared to placebo was reported [111] that would be consistent with reports of a tissue-specific suppression of endometrial prostaglandin production.

## 5. Conclusion

Studies on HMB are hampered by the lack of an appropriate *in vivo* model for fibroid-associated endometrial bleeding limiting our understanding of the interplay between fibroids and endometrium.

At present, the biggest challenge facing researchers who are keen to develop regimes using SPRMs as long-term treatments for women with debilitating benign gynecological conditions is a paucity of data related to the mechanisms underpinning the development of PAEC.

## 6. Expert opinion

Progesterone acting via its cognate receptors (PR-A, PR-B) plays a central role in regulation of uterine function making PR an attractive therapeutic target.

PRs classical genomic mode of action has been studied in detail and the impact of ligand binding, conformational change, and coactivator and -repressor recruitment on cell and tissue-specific patterns of gene expression is now better understood.

The regulation/impact of non-genomic progesterone pathways has recently been described as via the trigger of SRC-dependent phosphorylation signaling like proliferative RAS/RAF/MEKK/MAPK kinase pathway. The level of interaction and integration of these extranuclear signaling cascades with classical genomic signaling remains a topic for further studies.

A number of SPRMs have been developed which have a range of agonistic and antagonistic profiles when compared with progesterone. Mechanistic studies have identified distinct patterns of co-regulator recruitment that may in part explain their unique impact on cell function. Here, more molecular details will emerge in the future as the number of molecular studies on human tissues will increase as more SPRMs enter the market and it would be interesting to follow whether the systematic analysis of expression profiles of different fibroid and endometrial cellular components holds potential to generate a gene expression fingerprint which would be suitable to allow differentiation of SPRMs on their mechanistic action toward the different uterine cellular compartments.

Clinical studies indicate SPRMs hold promise for treatment of fibroids and associated HMB. Extensive *in vitro* studies on primary cells and small-scale investigations of biopsies from clinical studies highlighted inhibition of cell proliferation and an increase of the expression of proapoptotic markers and pathways and suppression of extracellular matrix synthesis as the molecular mechanism behind reduction of fibroid volume.

A link to progesterone action for fibroid growth has been offered by identification of distinct leiomyoma stem-progenitor cell populations processing paracrine signals from adjacent myometrium induced by progesterone. One of the key components mediating progesterone action in fibroid cells is the WNT/β-catenin pathway. Dissecting the paracrine mechanism involved with leiomyoma growth may also shed further light into the molecular action of SPRMs and may also lead to new treatments beyond SPRMs.

New fundamental insights into the etiology of fibroids will arise from the latest development in research regarding the MED12 mutation which is a driver mutation for fibroids with very high prevalence rate. Very recently, MED12-mutant mice have been generated, which will offer an opportunity to dissect downstream signaling of Med12 and investigate effects on tumor growth but also on AUB from adjacent endometrial tissue. Whether potential factors might be identified causative for induction of HMB will be an exciting area to follow.

In contrast to fibroid shrinkage, the cellular mechanisms by which SPRMs control endometrial bleeding are still poorly understood. Reduction in the numbers of uterine NK cells and their complex interaction with the spiral arteries and endometrial stroma cells maybe one possible explanation for SPRMs’ effect on bleeding suppression.

The administration of the SPRM class of compound is accompanied with morphological changes within the endometrium described as PAEC. These histological changes are recognized as a distinct histological entity and should not be confused with endometrial hyperplasia. However, whilst PAEC are well described and appear reversible, a greater understanding of molecular and cellular mechanisms underpinning these histological features is required.

## Article highlights

- Abnormal progesterone responses are implicated in a wide spectrum of benign human reproductive disorders, including abnormal uterine bleeding (AUB; including heavy menstrual bleeding [HMB]), fibroids (leiomyomas) and endometriosis.
- Progesterone acting via its cognate receptors (PR-A, PR-B) plays a central role in regulation of uterine function making PR an attractive therapeutic target.
- Differential recruitment of PR and associated transcriptional co-regulators to gene promoters are critical to tissue selective impacts of progesterone, for example, whereas it inhibits growth of the endometrium it stimulates growth of fibroids (leiomyomas).
- Selective progesterone receptor modulators (SPRMs) represent a new class of synthetic ligands, which can exert agonist, antagonist or mixed effects on various progesterone target tissues.
- Administration of all family members of the SPRM class of compound have been found to date to be accompanied with morphological changes within the endometrium described as progesterone receptor modulator associated endometrial changes (PAEC). These histological changes are recognized as a distinct histological entity.
- Whilst PAEC are now well described and appear reversible, the mechanisms by which these develop are poorly understood.

This box summarizes key points contained in the article.
